# A tutorial on the free-energy framework for modelling perception and learning

**DOI:** 10.1016/j.jmp.2015.11.003

**Published:** 2017-02

**Authors:** Rafal Bogacz

**Affiliations:** MRC Unit for Brain Network Dynamics, University of Oxford, Mansfield Road, Oxford, OX1 3TH, UK; Nuffield Department of Clinical Neurosciences, University of Oxford, John Radcliffe Hospital, Oxford, OX3 9DU, UK

## Abstract

This paper provides an easy to follow tutorial on the free-energy framework for modelling perception developed by Friston, which extends the predictive coding model of Rao and Ballard. These models assume that the sensory cortex infers the most likely values of attributes or features of sensory stimuli from the noisy inputs encoding the stimuli. Remarkably, these models describe how this inference could be implemented in a network of very simple computational elements, suggesting that this inference could be performed by biological networks of neurons. Furthermore, learning about the parameters describing the features and their uncertainty is implemented in these models by simple rules of synaptic plasticity based on Hebbian learning. This tutorial introduces the free-energy framework using very simple examples, and provides step-by-step derivations of the model. It also discusses in more detail how the model could be implemented in biological neural circuits. In particular, it presents an extended version of the model in which the neurons only sum their inputs, and synaptic plasticity only depends on activity of pre-synaptic and post-synaptic neurons.

## Highlights

• Bayesian inference about stimulus properties can be performed by networks of neurons.
• Learning about statistics of stimuli can be achieved by Hebbian synaptic plasticity.
• Structure of the model resembles the hierarchical organization of the neocortex.

## 1 Introduction

The model of Friston (2005) and the predictive coding model of Rao and Ballard (1999) provide a powerful mathematical framework to describe how the sensory cortex extracts information from noisy stimuli. The predictive coding model (Rao & Ballard, 1999) suggests that visual cortex infers the most likely properties of stimuli from noisy sensory input. The inference in this model is implemented by a surprisingly simple network of neuron-like nodes. The model is called “predictive coding”, because some of the nodes in the network encode the differences between inputs and predictions of the network. Remarkably, learning about features present in sensory stimuli is implemented by simple Hebbian synaptic plasticity, and Rao and Ballard (1999) demonstrated that the model presented with natural images learns features resembling receptive fields of neurons in the primary visual cortex.

Friston (2005) has extended the model to also represent uncertainty associated with different features. He showed that learning about the variance and co-variance of features can also be implemented by simple synaptic plasticity rules based on Hebbian learning. As the extended model (Friston, 2005) learns the variance and co-variance of features, it offers several new insights. First, it describes how the perceptual systems may differentially weight sources of sensory information depending on their level of noise. Second, it shows how the sensory networks can learn to recognize features that are encoded in the patterns of covariance between inputs, such as textures. Third, it provides a natural way to implement attentional modulation as the reduction in variance of the attended features (we come back to these insights in Discussion). Furthermore, Friston (2005) pointed out that this model can be viewed as an approximate Bayesian inference based on minimization of a function referred to in statistics as free-energy. The free-energy framework (Friston, 2003, Friston, 2005) has been recently extended by Karl Friston and his colleagues to describe how the brain performs different cognitive functions including action selection (FitzGerald et al., 2015, Friston et al., 2013). Furthermore, Friston (2010) proposed that the free-energy theory unifies several theories of perception and action which are closely related to the free-energy framework.

There are many articles which provide an intuition for the free-energy framework and discuss how it relates with other theories and experimental data (Friston, 2003, Friston, 2005, Friston, 2010, Friston et al., 2013). However, the description of mathematical details of the theory in these papers requires a very deep mathematical background. The main goal of this paper is to provide an easy to follow tutorial on the free-energy framework. To make the tutorial accessible to a wide audience, it only assumes basic knowledge of probability theory, calculus and linear algebra. This tutorial is planned to be complementary to existing literature so it does not focus on the relationship to other theories and experimental data, and on applications to more complex tasks which are described elsewhere (Friston, 2010, Friston et al., 2013).

In this tutorial we also consider in more detail the neural implementation of the free-energy framework. Any computational model would need to satisfy the following constraints to be considered biologically plausible:

1. Local computation: A neuron performs computations only on the basis of the activity of its input neurons and synaptic weights associated with these inputs (rather than information encoded in other parts of the circuit).
2. Local plasticity: Synaptic plasticity is only based on the activity of pre-synaptic and post-synaptic neurons.

The model of Rao and Ballard (1999) fully satisfied these constraints. The model of Friston (2005) did not satisfy them fully, but we show that after small modifications and extensions it can satisfy them. So the descriptions of the model in this tutorial slightly differ in a few places or extend the original model to better explain how the proposed computation could be implemented in the neural circuits. All such differences or extensions are indicated by footnotes or in text, and the original model is presented in Appendix A.

It is commonly assumed in theoretical neuroscience, (O’Reilly & Munakata, 2000) that the basic computations a neuron performs are the summation of its input weighted by the strengths of synaptic connections, and the transformation of this sum through a (monotonic) function describing the relationship between neurons’ total input and output (also termed firing-Input or f-I curve). Whenever possible, we will assume that the computation of the neurons in the described model is limited to these computations (or even just to linear summation of inputs).

We feel that the neural implementation of the model is worth considering, because if the free-energy principle indeed describes the computations in the brain, it can provide an explanation for why the cortex is organized in a particular way. However to gain such insight it is necessary to start comparing the neural networks implementing the model with those in the real brain. Consequently, we consider in this paper possible neural circuits that could perform the computations required by the theory. Although the neural implementations proposed here are not the only possible ones, it is worth considering them as a starting point for comparison of the model with details of neural architectures in the brain. We hope that such comparison could iteratively lead to refined neural implementations that are more and more similar to real neural circuits.

To make this tutorial as easy to follow as possible we introduce the free-energy framework using a simple example, and then illustrate how the model can scale up to more complex neural architectures. The tutorial provides step-by-step derivation of the model. Some of these derivations are straightforward, and we feel that it would be helpful for the reader to do them on their own to gain a better understanding of the model and to “keep in mind” the notation used in the paper. Such straightforward derivations are indicated by “(TRY IT YOURSELF)”, so after encountering such label we recommend trying to do the calculation described in the sentence with this label and then compare the obtained results with those in the paper. To illustrate the model we include simple simulations, but again we feel it would be helpful for a reader to perform them on their own, to get an intuition for the model. Therefore we describe these simulations as exercises.

The paper is organized as follows. Section  2 introduces the model using a very simple example using as basic mathematical concepts as possible, so it is accessible to a particularly wide audience. Section  3 provides mathematical foundations for the model, and shows how the inference in the model is related to minimization of free-energy. Section  4 then shows how the model scales up to describe the neural circuits in sensory cortex. In these three sections we use notation similar to that used by Friston (2005). Section  5 describes an extended version of the model which satisfies the constraint of local plasticity described above. Finally, Section  6 discusses insights provided by the model.

## 2 Simplest example of perception

We start by considering in this section a simple perceptual problem in which a value of a single variable has to be inferred from a single observation. To make it more concrete, consider a simple organism that tries to infer the size or diameter of a food item, which we denote by v, on the basis of light intensity it observes. Let us assume that our simple animal has only one light sensitive receptor which provides it with a noisy estimate of light intensity, which we denote by u. Let g denote a non-linear function relating the average light intensity with the size. Since the amount of light reflected is related to the area of an object, in this example we will consider a simple function of $g\left(v\right)=v^{2}$. Let us further assume that the sensory input is noisy—in particular, when the size of food item is v, the perceived light intensity is normally distributed with mean g(v), and variance $\Sigma_{u}$ (although a normal distribution is not the best choice for a distribution of light intensity, as it includes negative numbers, we will still use it for a simplicity):

(1) $$p\left(u|v\right)=f\left(u;g\left(v\right),\Sigma_{u}\right)\text{.}$$

In Eq. (1)f(x;μ,Σ) denotes the density of a normal distribution with mean μ and variance Σ:

(2) $$f\left(x;\mu ,\Sigma \right)=\frac{1}{\sqrt{2\pi \Sigma }}\exp\left(-\frac{{\left(x-\mu \right)}^{2}}{2\Sigma }\right)\text{.}$$

Due to the noise present in the observed light intensity, the animal can refine its guess for the size v by combining the sensory stimulus with the prior knowledge on how large the food items usually are, that it had learnt from experience. For simplicity, let us assume that our animal expects this size to be normally distributed with mean $v_{p}$ and variance $\Sigma_{p}$ (subscript p stands for “prior”), which we can write as:

(3) $$p\left(v\right)=f\left(v;v_{p},\Sigma_{p}\right)\text{.}$$

Let us now assume that our animal observed a particular value of light intensity, and attempts to estimate the size of the food item on the basis of this observation. We will first consider an exact solution to this problem, and illustrate why it would be difficult to compute it in a simple neural circuit. Then we will present an approximate solution that can be easily implemented in a simple network of neurons.

### 2.1 Exact solution

To compute how likely different sizes v are given the observed sensory input u, we could use Bayes’ theorem:

(4) $$p\left(v|u\right)=\frac{p\left(v\right)p\left(u|v\right)}{p\left(u\right)}\text{.}$$

Term p(u) in the denominator of Eq. (4) is a normalization term, which ensures that the posterior probabilities of all sizes p(v|u) integrate to 1:

(5) p(u)=∫p(v)p(u|v)dv.

The integral in the above equation sums over the whole range of possible values of v, so it is a definite integral, but for brevity of notation we do not state the limits of integration in this and all other integrals in the paper.

Now combining Eqs. (1), (2), (3), (4), (5) we can compute numerically how likely different sizes are given the sensory observation. For readers who are not familiar with such Bayesian inference we recommend doing the following exercise now.

Exercise 1 *Assume that our animal observed the light intensity* u=2*, the level of noise in its receptor is* $\Sigma_{u}=1$*, and the mean and variance of its prior expectation of size are* $v_{p}=3$ *and* $\Sigma_{p}=1$*. Write a computer program that computes the posterior probabilities of sizes from*  0.01  *to*  5*, and plots them.*

**Fig. 1:**
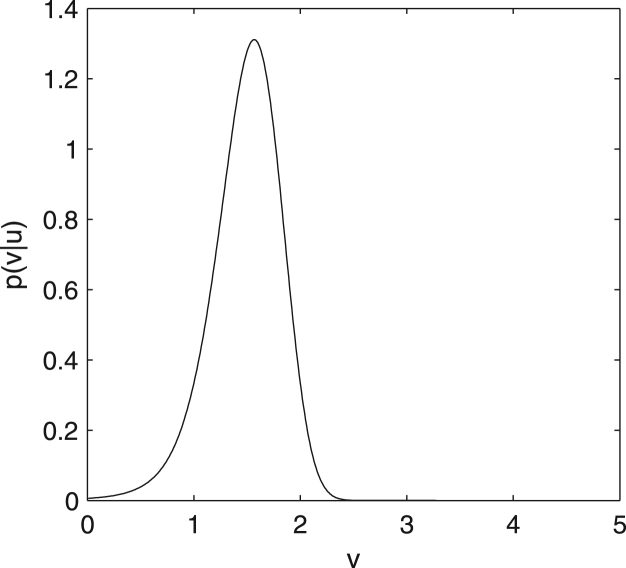
The posterior probability of the size of the food item in the problem given in Exercise 1.

The Matlab code performing this calculation is given at the end of the paper, and the resulting plot is shown in Fig. 1. It is worth observing that such Bayesian approach integrates the information brought by the stimulus with prior knowledge: please note that the most likely value of v lies between that suggested by the stimulus (i.e. $\sqrt{2}$) and the most likely value based on prior knowledge (i.e. 3). It may seem surprising why the posterior probability is so low for v=3, i.e. the mean prior expectation. It comes from the fact that g(3)=9, which is really far from observed value u=2, so p(u=2|v=3) is very close to zero. This illustrates how non-intuitive Bayesian inference can be once the relationship between variables is non-linear.

Let us now discuss why performing such exact calculation is challenging for a simple biological system. First, as soon as function g relating the variable we wish to infer with observations is non-linear, the posterior distribution p(v|u) may not take a standard shape—for example the distribution in Fig. 1 is not normal. Thus representing the distribution p(v|u) requires representing infinitely many values p(v|u) for different possible u rather than a few summary statistics like mean and variance. Second, the computation of the posterior distribution involves computation of the normalization term. Although it has been proposed that circuits within the basal ganglia can compute the normalization term in case of the discrete probability distributions (Bogacz & Gurney, 2007), computation of the normalization for continuous distributions involves evaluating the integral of Eq. (5). Calculating such integral would be challenging for a simple biological system. This is especially true when the dimensionality of the integrals (i.e., the number of unknown variables) increases beyond a trivial number. Even mathematicians resort to (computationally very expensive) numerical or sampling techniques in this case.

We will now present an approximate solution to the above inference problem, that could be easily implemented in a simple biological system.

### 2.2 Finding the most likely feature value

Instead of finding the whole posterior distribution p(v|u), let us try to find the most likely size of the food item v which maximizes p(v|u). We will denote this most likely size by ϕ, and its posterior probability density by p(ϕ|u). It is reasonable to assume that in many cases the brain represents at a given moment of time only most likely values of features. For example in case of binocular rivalry, only one of the two possible interpretations of sensory inputs is represented.

We will look for the value ϕ which maximizes p(ϕ|u). According to Eq. (4), the posterior probability p(ϕ|u) depends on a ratio of two quantities, but the denominator p(u) does not depend on ϕ. Thus the value of ϕ which maximizes p(ϕ|u) is the same one which maximizes the numerator of Eq. (4). We will denote the logarithm of the numerator by F, as it is related to the negative of free energy (as we will describe in Section  3):

(6) F=lnp(ϕ)+lnp(u|ϕ).

In the above equation we used the property of logarithm ln(ab)=lna+lnb. We will maximize the logarithm of the numerator of Eq. (4), because it has the same maximum as the numerator itself as ln is a monotonic function, and is easier to compute as the expressions for p(u|ϕ) and p(ϕ) involve exponentiation.

To find the parameter ϕ that describes the most likely size of the food item, we will use a simple gradient ascent: i.e. we will modify ϕ proportionally to the gradient of F, which will turn out to be a very simple operation. It is relatively straightforward to compute F by substituting Eqs. (1), (2), (3) into Eq. (6) and then to compute the derivative of F (TRY IT YOURSELF).

(7) $$F=\ln f\left(\phi ;v_{p},\Sigma_{p}\right)+\ln f\left(u;g\left(\phi \right),\Sigma_{u}\right)=\ln\left[\frac{1}{\sqrt{2\pi \Sigma_{p}}}\exp\left(-\frac{{\left(\phi -v_{p}\right)}^{2}}{2\Sigma_{p}}\right)\right]+\ln\left[\frac{1}{\sqrt{2\pi \Sigma_{u}}}\exp\left(-\frac{{\left(u-g\left(\phi \right)\right)}^{2}}{2\Sigma_{u}}\right)\right]=\ln\frac{1}{\sqrt{2\pi }}-\frac{1}{2}\ln\Sigma_{p}-\frac{{\left(\phi -v_{p}\right)}^{2}}{2\Sigma_{p}}+\ln\frac{1}{\sqrt{2\pi }}-\frac{1}{2}\ln\Sigma_{u}-\frac{{\left(u-g\left(\phi \right)\right)}^{2}}{2\Sigma_{u}}=\frac{1}{2}\left(-\ln\Sigma_{p}-\frac{{\left(\phi -v_{p}\right)}^{2}}{\Sigma_{p}}-\ln\Sigma_{u}-\frac{{\left(u-g\left(\phi \right)\right)}^{2}}{\Sigma_{u}}\right)+C\text{.}$$

We incorporated the constant terms in the 2nd line above into a constant C. Now we can compute the derivative of F over ϕ:

(8) $$\frac{\partial F}{\partial \phi }=\frac{v_{p}-\phi }{\Sigma_{p}}+\frac{u-g\left(\phi \right)}{\Sigma_{u}}g^{\prime }\left(\phi \right)\text{.}$$

In the above equation we used the chain rule to compute the second term, and $g^{\prime }\left(\phi \right)$ is a derivative of function g evaluated at ϕ, so in our example $g^{\prime }\left(\phi \right)=2\phi$. We can find our best guess ϕ for v simply by changing ϕ in proportion to the gradient:

(9) $$\overset{˙}{\phi }=\frac{\partial F}{\partial \phi }\text{.}$$

In the above equation $\overset{˙}{\phi }$ is the rate of change of ϕ with time. Let us note that the update of ϕ is very intuitive. It is driven by two terms in Eq. (8): the first moves it towards the mean of the prior, the second moves it according to the sensory stimulus, and both terms are weighted by the reliabilities of prior and sensory input respectively.

Now please note that the above procedure for finding the approximate distribution of distance to food item is computationally much simpler than the exact method presented at the start of the paper. To gain more appreciation for the simplicity of this computation we recommend doing the following exercise.

Exercise 2 *Write a computer program finding the most likely size of the food item* ϕ *for the situation described in*   Exercise  1*. Initialize* $\phi =v_{p}$*, and then find its values in the next*  5  *time units (you can use Euler’s method, i.e. update* ϕ(t+Δt)=ϕ(t)+Δt∂F/∂ϕ *with* Δt=0.01 *).*

**Fig. 2:**
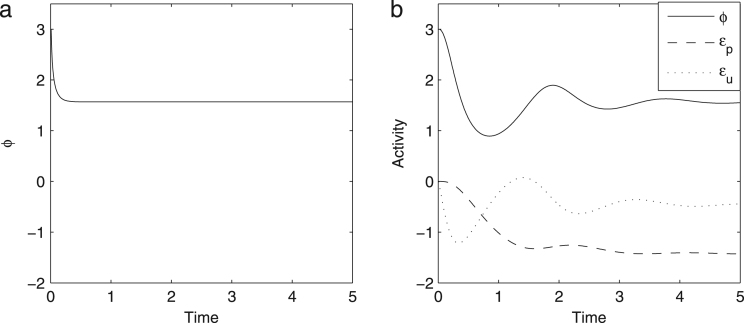
Solutions to Exercise 2, Exercise 3. In panel b we have also included quantities that we will see later can be regarded as prediction errors.

Fig. 2(a) shows a solution to Exercise 2. Please notice that it rapidly converges to the value of ϕ≈1.6, which is also the value that maximizes the exact posterior probability p(v|u) shown in Fig. 1.

### 2.3 A possible neural implementation

One can envisage many possible ways in which the computation described in previous subsection could be implemented in neural circuits. In this paper we will present a possible implementation which satisfies the constraints of local computation and plasticity described in the Introduction. It slightly differs from the original implementation which is contained in Appendix A.

While thinking about the neural implementation of the above computation, it is helpful to note that there are two similar terms in Eq. (8), so let us denote them by new variables.

(10) $$\varepsilon_{p}=\frac{\phi -v_{p}}{\Sigma_{p}}$$

(11) $$\varepsilon_{u}=\frac{u-g\left(\phi \right)}{\Sigma_{u}}\text{.}$$

The above terms are the prediction errors^1^: $\varepsilon_{u}$ expresses how much the light intensity differs from that expected if the size of the food item was ϕ, while $\varepsilon_{p}$ denotes how the inferred size differs from prior expectations. With these new variables the equation for updating ϕ simplifies to:

(12) $$\overset{˙}{\phi }=\varepsilon_{u}g^{\prime }\left(\phi \right)-\varepsilon_{p}\text{.}$$

The neural implementation of the model assumes that the model parameters $v_{p}$, $\Sigma_{p}$, and $\Sigma_{u}$ are encoded in the strengths of synaptic connections (as they need to be maintained over the animal’s lifetime), while variables ϕ, $\varepsilon_{u}$, and $\varepsilon_{p}$ and the sensory input u are maintained in the activity of neurons or neuronal populations (as they change rapidly when the sensory input is modified). In particular, we will consider very simple neural “nodes” which simply change their activity proportionally to the input they receive, so for example, Eq. (12) is implemented in the model by a node receiving input equal to the right hand side of this equation. The prediction errors could be computed by the nodes with the following dynamics^2^:

(13) $$\overset{˙}{\varepsilon_{p}}=\phi -v_{p}-\Sigma_{p}\varepsilon_{p}$$

(14) $$\overset{˙}{\varepsilon_{u}}=u-g\left(\phi \right)-\Sigma_{u}\varepsilon_{u}\text{.}$$

It is easy to show that the nodes with dynamics described by Eqs. (13)–(14) converge to the values defined in Eqs. (10)–(11). Once Eqs. (13)–(14) converge, then $\overset{˙}{\varepsilon }=0$, so setting $\overset{˙}{\varepsilon }=0$ and solving Eqs. (13)–(14) for ε, one obtains Eqs. (10)–(11).

**Fig. 3:**
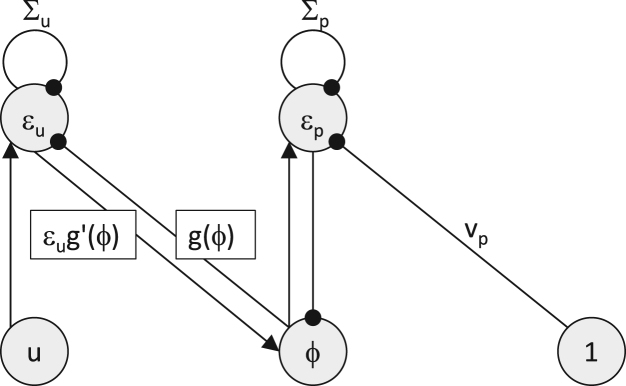
The architecture of the model performing simple perceptual inference. Circles denote neural “nodes”, arrows denote excitatory connections, while lines ended with circles denote inhibitory connections. Labels above the connections encode their strength, and lack of label indicates the strength of 1. Rectangles indicate the values that need to be transmitted via the connections they label.

The architecture of the network described by Eqs. (12), (13), (14) is shown in Fig. 3. Let us consider the computations in its nodes. The node $\varepsilon_{p}$ receives excitatory input from node ϕ, inhibitory input from a tonically active neuron via a connection with strength $v_{p}$, and inhibitory input from itself via a connection with strength $\Sigma_{p}$, so it implements Eq. (13). The nodes ϕ and $\varepsilon_{u}$ analogously implement Eqs. (12), (14), but here the information exchange between them is additionally affected by function g, and we will discuss this issue in more detail in Section  2.5. We have now described all the details necessary to simulate the model.

Exercise 3 *Simulate the model from*   Fig.  3   *for the problem from*   Exercise  1*. In particular, initialize* $\phi =v_{p},\;\varepsilon_{p}=\varepsilon_{u}=0$*, and find their values for the next*  5  *units of time.*

Solution to Exercise 3 is shown in Fig. 2(b). The model converges to the same value as in Fig. 2(a), but the convergence is just slower, as the model now includes multiple nodes connected by excitatory and inhibitory connections and such networks have oscillatory tendencies, so these oscillations need to settle for the network to converge.

### 2.4 Learning model parameters

As our imaginary animal perceives food items through its lifetime, it may wish to refine its expectation about typical sizes of food items described by parameters $v_{p}$ and $\Sigma_{p}$, and about the amount of error it makes observing light intensity, described by parameter $\Sigma_{u}$. Thus it may wish to update the parameters $v_{p},\;\Sigma_{p}$, and $\Sigma_{u}$ after each stimulus to gradually refine them.

We wish to choose the model parameters for which the perceived light intensities u are least surprising, or in other words most expected. Thus we wish to choose parameters that maximize p(u). However, please recall that p(u) is described by a complicated integral of Eq. (5), so it would be difficult to maximize p(u) directly. Nevertheless, it is simple to maximize a related quantity p(u,ϕ), which is the joint probability of sensory input u and our inferred food size ϕ. Note that p(u,ϕ)=p(ϕ)p(u|ϕ), so F=lnp(u,ϕ), thus maximization of p(u,ϕ) can be achieved by maximizing F. A more formal explanation for why the parameters can be optimized by maximizing F will be provided in Section  3.

The model parameters can be hence optimized by modifying them proportionally to the gradient of F. Starting with the expression in Eq. (7) it is straightforward to find the derivatives of F over $v_{p},\;\Sigma_{p}$ and $\Sigma_{u}$ (TRY IT YOURSELF):

(15) $$\frac{\partial F}{\partial v_{p}}=\frac{\phi -v_{p}}{\Sigma_{p}}$$

(16) $$\frac{\partial F}{\partial \Sigma_{p}}=\frac{1}{2}\left(\frac{{\left(\phi -v_{p}\right)}^{2}}{\Sigma_{p}^{2}}-\frac{1}{\Sigma_{p}}\right)$$

(17) $$\frac{\partial F}{\partial \Sigma_{u}}=\frac{1}{2}\left(\frac{{\left(u-g\left(\phi \right)\right)}^{2}}{\Sigma_{u}^{2}}-\frac{1}{\Sigma_{u}}\right)\text{.}$$

Let us now provide an intuition for why the parameter update rules have their particular form. We note that since parameters are updated after observing each food item, and different food items observed during animal’s life time have different sizes, the parameters never converge. Nevertheless it is useful to consider the values of parameters for which the expected value of change is 0, as these are the values in vicinity of which the parameters are likely to be. For example, according to Eq. (15), the expected value of change in $v_{p}$ is 0 when $\langle \left(\phi -v_{p}\right)/\Sigma_{p}\rangle =0$, where 〈〉 denotes the expected value over trials. This will happen if $v_{p}=\langle \phi \rangle$, i.e. when $v_{p}$ is indeed equal to the expected value of ϕ. Analogously, the expected value of change in $\Sigma_{p}$ is 0 when:

(18) $$\langle \frac{{\left(\phi -v_{p}\right)}^{2}}{\Sigma_{p}^{2}}-\frac{1}{\Sigma_{p}}\rangle =0\text{.}$$

Rearranging the above condition one obtains $\Sigma_{p}=\langle {\left(\phi -v_{p}\right)}^{2}\rangle$, thus the expected value of change in $\Sigma_{p}$ is 0, when $\Sigma_{p}$ is equal to the variance of ϕ. An analogous analysis can be made for $\Sigma_{u}$.

Eqs. (15), (16), (17) for update of model parameters simplify significantly when they are written in terms of prediction errors (TRY IT YOURSELF):

(19) $$\frac{\partial F}{\partial v_{p}}=\varepsilon_{p}$$

(20) $$\frac{\partial F}{\partial \Sigma_{p}}=\frac{1}{2}\left(\varepsilon_{p}^{2}-\Sigma_{p}^{-1}\right)$$

(21) $$\frac{\partial F}{\partial \Sigma_{u}}=\frac{1}{2}\left(\varepsilon_{u}^{2}-\Sigma_{u}^{-1}\right)\text{.}$$

The above rules for update of parameters correspond to very simple synaptic plasticity mechanisms. All rules include only values that can be “known” by the synapse, i.e. the activities of pre-synaptic and post-synaptic neurons, and the strengths of the synapse itself. Furthermore, the rules are Hebbian, in the sense that they depend on the products of activity of pre-synaptic and post-synaptic neurons. For example, the change in $v_{p}$ in Eq. (19) is equal to the product of pre-synaptic activity (i.e. 1) and the post-synaptic activity $\varepsilon_{p}$. Similarly, the changes in Σ in Eqs. (20)–(21) depend on the products of pre-synaptic and post-synaptic activities, both equal to ε.

The plasticity rules of Eqs. (20)–(21) also depend on the value of synaptic weights themselves, as they include terms $\Sigma^{-1}$. For the simple case considered in this section, the synapse “has access” to the information on its weight. Moreover, the dependence of synaptic plasticity on initial weights has been seen experimentally (Chen et al., 2013), so we feel it is plausible for the dependence predicted by the model to be present in real synapses. However, when the model is scaled up to include multiple features and sensory inputs in Section  4.1, terms $\Sigma^{-1}$ will turn into a matrix inverse (in Eqs. (48)–(49)), so the required changes in each weight will depend on the weights of other synapses in the network. Nevertheless, we will show in Section  5 how this problem can be overcome.

Finally, we would like to discuss the limits on parameters Σ. Although in principle the variance of a random variable can be equal to 0, if $\Sigma_{p}=0$ or $\Sigma_{u}=0$, then Eq. (13) or (14) would not converge but instead $\varepsilon_{p}$ or $\varepsilon_{u}$ would diverge to positive or negative infinity. Similarly, if Σ were close to 0, the convergence would be very slow. To prevent this from happening, the minimum value of 1 is imposed by Friston (2005) on the estimated variance.^3^

### 2.5 Learning the relationship between variables

So far we have assumed for simplicity that the relationship g between the variable being inferred and the stimulus is known. However, in general it may not be known, or may need to be tuned. So we will now consider function g(v,θ) that also depends on parameter which we denote by θ.

We will consider two special cases of function g(v,θ), where the parameter θ has a clear biological interpretation. First, let us consider a simple case of a linear function: g(v,θ)=θv, as then the model has a straightforward neural implementation. In this case, Eqs. (12), (13), (14) describing the model simplify to:

(22) $$\overset{˙}{\phi }=\theta \varepsilon_{u}-\varepsilon_{p}$$

(23) $$\overset{˙}{\varepsilon_{p}}=\phi -v_{p}-\Sigma_{p}\varepsilon_{p}$$

(24) $$\overset{˙}{\varepsilon_{u}}=u-\theta \phi -\Sigma_{u}\varepsilon_{u}$$

**Fig. 4:**
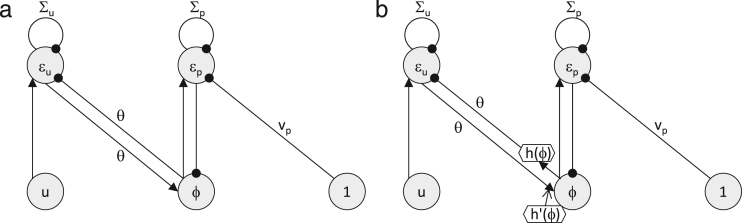
Architectures of models with linear and nonlinear function g. Circles and hexagons denote linear and nonlinear nodes respectively. Filled arrows and lines ended with circles denote excitatory and inhibitory connections respectively, and an open arrow denotes a modulatory influence.

In this model, nodes ϕ and ε simply communicate through connections with weight θ as shown in Fig. 4(a). Furthermore, we can also derive the rule for updating the parameter θ by finding the gradient of F over θ, as now function g in Eq. (7) depends on θ (TRY IT YOURSELF):

(25) $$\frac{\partial F}{\partial \theta }=\varepsilon_{u}\phi \text{.}$$

Please note that this rule is again Hebbian, as the synaptic weights encoding θ are modified proportionally to the activities of pre-synaptic and post-synaptic neurons (see Fig. 4(a)).

Second, let us consider a case of a nonlinear function^4^g(v,θ)=θh(v), where h(v) is a nonlinear function that just depends on v, as it results in only slightly more complex neural implementation. Furthermore, this situation is relevant to the example of the simple animal considered at the start of this section, as the light is proportional to the area, but the proportionality constant may not be known (this case is also relevant to the network that we will discuss in Section  4.1). In this case, Eqs. (12), (13), (14) describing the model become:

(26) $$\overset{˙}{\phi }=\theta \varepsilon_{u}h^{\prime }\left(\phi \right)-\varepsilon_{p}$$

(27) $$\overset{˙}{\varepsilon_{p}}=\phi -v_{p}-\Sigma_{p}\varepsilon_{p}$$

(28) $$\overset{˙}{\varepsilon_{u}}=u-\theta h\left(\phi \right)-\Sigma_{u}\varepsilon_{u}\text{.}$$

A possible network implementing this model is illustrated in Fig. 4(b), which now includes non-linear elements. In particular, the node ϕ sends to node $\varepsilon_{u}$ its activity transformed by a non-linear function, i.e. θh(ϕ). One could imagine that this could be implemented by an additional node receiving input from node ϕ, transforming it via a non-linear transformation h and sending its output to node $\varepsilon_{u}$ via a connection with the weight θ. Analogously, the input from node $\varepsilon_{u}$ to node ϕ needs to be scaled by $\theta h^{\prime }\left(\phi \right)$. Again one could imagine that this could be implemented by an additional node receiving input from node ϕ, transforming it via a non-linear transformation $h^{\prime }$ and modulating input received from node $\varepsilon_{u}$ via a connection with weight θ (alternatively, this could be implemented within the node ϕ by making it react to its input differentially depending on its level of activity). The details of the neural implementation of these non-linear transformations depend on the form of function h, and would be an interesting direction of the future work.

We also note that the update of the parameter θ, i.e. gradient of F over θ becomes:

(29) $$\frac{\partial F}{\partial \theta }=\varepsilon_{u}h\left(\phi \right)\text{.}$$

This rule is Hebbian for the top connection labelled by θ in Fig. 4(b), as it is a product of activity of the pre-synaptic and post-synaptic nodes. It would be interesting to investigate how such a plasticity rule could be realized for the other connection with the weight of θ (from node $\varepsilon_{u}$ to ϕ). We just note that for this connection the rule also satisfies the constraint of local plasticity (stated in the Introduction), as ϕ fully determines h(ϕ), so the change in weight is fully determined by the activity of pre-synaptic and post-synaptic neurons.

## 3 Free-energy

In this section we discuss how the computations in the model relate to a technique of statistical inference involving minimization of free-energy. There are three reasons for describing this relationship. First, it will provide more insight for why the parameters can be optimized by maximization of F. Second, the concept of free-energy is critical for understanding of more complex models (Friston et al., 2013), which not only estimate the most likely values of variables, but their distribution. Third, the free-energy is a very interesting concept on its own, and has applications in mathematical psychology (Ostwald, Kirilina, Starke, & Blankenburg, 2014).

We now come back to the example of an inference by a simple organism, and discuss how the exact inference described in Section  2.1 can be approximated. As we noted in Section  2.1, the posterior distribution p(v|u) may have a complicated shape, so we will approximate it with another distribution, which we denote q(v). Importantly, we will assume that q(v) has a standard shape, so we will be able to characterize it by parameters of this typical distribution. For example, if we assume that q(v) is normal, then to fully describe it, we can infer just two numbers: its mean and variance, instead of infinitely many numbers potentially required to characterize a distribution of an arbitrary shape.

For simplicity, here we will use an even simpler shape of the approximate distribution, namely the delta distribution, which has all its mass cumulated in one point which we denote by ϕ (i.e. the delta distribution is equal to 0 for all values different from ϕ, but its integral is equal to 1). Thus we will try to infer from observation just one parameter ϕ which will characterize the most likely value of v.

We now describe what criterion we wish our approximate distribution to satisfy. We will seek the approximate distribution q(v) which is as close as possible to the actual posterior distribution p(v|u). Mathematically, the dissimilarity between two distributions in measured by the Kullback–Leibler divergence defined as:

(30) $$\mathrm{KL}\left(q\left(v\right),p\left(v|u\right)\right)=\int q\left(v\right)\ln\frac{q\left(v\right)}{p\left(v|u\right)}dv\text{.}$$

For readers not familiar with Kullback–Leibler divergence we would like clarify why it is a measure of dissimilarity between the distributions. Please note that if the two distributions q(v) and p(v|u) were identical, the ratio q(v)/p(v|u) would be equal to 1, so its logarithm would be equal to 0, and so the whole expression in Eq. (30) would be 0. The Kullback–Leibler divergence also has a property that the more different the two distributions are, the higher its value is (see Ostwald et al. (2014) for more details).

Since we assumed above that our simplified distribution is a delta function, we will simply seek the value of its centre parameter ϕ which minimizes the Kullback–Leibler divergence defined in Eq. (30).

It may seem that the minimization of Eq. (30) is still difficult, because to compute term p(v|u) present in Eq. (30) from Bayes’ theorem (Eq. (4)) one needs to compute the difficult normalization integral (Eq. (5)). However, we will now show that there exists another way of finding the approximate distribution q(v) that does not involve the complicated computation of the normalization integral.

Substituting the definition of conditional probability p(v|u)=p(u,v)/p(u) into Eq. (30) we obtain:

(31) $$\mathrm{KL}\left(q\left(v\right),p\left(v|u\right)\right)=\int q\left(v\right)\ln\frac{q\left(v\right)p\left(u\right)}{p\left(u,v\right)}dv=\int q\left(v\right)\ln\frac{q\left(v\right)}{p\left(u,v\right)}dv+\int q\left(v\right)dv\ln p\left(u\right)=\int q\left(v\right)\ln\frac{q\left(v\right)}{p\left(u,v\right)}dv+\ln p\left(u\right)\text{.}$$

In the transition from the second to the third line we used the fact that q(v) is a probability distribution so its integral is 1. The integral in the last line of the above equation is called free-energy, and we will denote its negative by F, because we will show below, that for certain assumptions the negative free-energy is equal (modulo a constant) to the function F we defined and used in the previous section:

(32) $$F=\int q\left(v\right)\ln\frac{p\left(u,v\right)}{q\left(v\right)}dv\text{.}$$

In the above equation we used the property of logarithms that −lna/b=lnb/a. So, the negative free-energy is related to the Kullback–Leibler divergence in the following way:

(33) KL(q(v),p(v|u))=−F+lnp(u).

Now please note that lnp(u) does not depend on ϕ (which is a parameter describing q(v)), so the value of ϕ that minimizes the distance between q(v) and p(v|u) is the same value as that which maximizes F. Therefore instead of minimizing the Kullback–Leibler divergence we can maximize F, and this will have two benefits: first, as we already mentioned above, F is easier to compute as it does not involve the complicated computation of the normalization term. Second, as we will see later, it will allow us to naturally introduce learning about the parameters of the model.

Let us first note that by assuming that q(v) is a delta distribution, the negative free energy simplifies to:

(34) $$F=\int q\left(v\right)\ln\frac{p\left(u,v\right)}{q\left(v\right)}dv=\int q\left(v\right)\ln p\left(u,v\right)dv-\int q\left(v\right)\ln q\left(v\right)dv=\ln p\left(u,\phi \right)+C_{1}\text{.}$$

In the transition from the first to the second line above we used the property of logarithms ln(a/b)=lna−lnb. In the transition from the second line to the third line we used the property of a delta function δ(x) with centre ϕ that for any function h(x), the integral of δ(x)h(x) is equal to h(ϕ). Furthermore, since the value of the second integral in the second line of the above equation does not depend on ϕ (so it will cancel when we compute the derivative over ϕ) we denote it by a constant $C_{1}$.

Now using p(u,ϕ)=p(ϕ)p(u|ϕ), and ignoring constant $C_{1}$, we obtain the expression for F we introduced previously in Eq. (6). Thus finding approximate delta distribution q(v) through minimization of free-energy is equivalent to the inference of features in the model described in the previous section. It is worth noting that Eq. (34) states that the best centre for our approximate distribution (i.e. our best guess for the size of the food item) is the value v=ϕ which maximizes the joint probability p(u,ϕ).

We now discuss how the concept of free-energy will help us to understand why the parameters of the model can be learnt by maximization of F. Recall from Section  2.4 that we wish to find parameters for which the sensory observations are least surprising, i.e. those which maximize p(u). To see the relationship between maximizing p(u) and maximizing F, we note that according to Eq. (33), p(u) is related to the negative free-energy in the following way:

(35) lnp(u)=F+KL(q(v),p(v|u)).

Since Kullback–Leibler divergence is non-negative, F is a lower bound on lnp(u), thus by maximizing F we maximize the lower bound on lnp(u). So in summary, by maximizing F we can both find an approximate distribution q(v) (as discussed earlier), and optimize model parameters. However, there is a twist here: we wish to maximize the average of p(u) across trials (or here observations of different food items). Thus on each trial we need to modify the model parameters just a little bit (rather than until minimum of free energy is reached as was the case for ϕ).

## 4 Scaling up the model of perception

In this section we will show how the model scales up to the networks inferring multiple features and involving hierarchy.

### 4.1 Increasing the dimension of sensory input

The model naturally scales up to the case of multiple sensory inputs from which we estimate multiple variables. Such scaled model could be used to describe information processing within a cortical area (e.g. primary visual cortex) which infers multiple features (e.g. edges at different position and orientation) on the basis of multiple inputs (e.g. information from multiple retinal receptors preprocessed by the thalamus). This section shows that when the dimensionality of inputs and features is increased, the dynamics of nodes in the networks and synaptic plasticity are described by the same rules as in Section  2, just generalized to multiple dimensions.

The only complication in explaining this case lies in the necessity to use matrix notation, so let us make this notation very explicit: we will denote single numbers in italic (e.g. x), column vectors by bar (e.g. $\bar{x}$), and matrices in bold (e.g. x). So we assume the animal has observed sensory input $\bar{u}$ and estimates the most likely values $\bar{\phi }$ of variables $\bar{v}$. We further assume that the animal has prior expectation that the variables $\bar{v}$ come from multivariate normal distribution with mean ${\bar{v}}_{p}$ and covariance matrix $\Sigma_{p}$, i.e. $p\left(\bar{v}\right)=f\left(\bar{v};{\bar{v}}_{p},\Sigma_{p}\right)$ where:

(36) $$f\left(\bar{x};\bar{\mu },\Sigma \right)=\frac{1}{\sqrt{{\left(2\pi \right)}^{N}\left|\Sigma \right|}}\exp\left(-\frac{1}{2}{\left(\bar{x}-\bar{\mu }\right)}^{T}\Sigma^{-1}\left(\bar{x}-\bar{\mu }\right)\right)\text{.}$$

In the above equation N denotes the length of vector $\bar{x}$, and |Σ| denotes the determinant of matrix Σ. Analogously, the probability of observing sensory input given the values of variables is given by $p\left(\bar{u}|\bar{v}\right)=f\left(\bar{u};g\left(\bar{v},\Theta \right),\Sigma_{u}\right)$, where Θ are parameters of function g. We denote these parameters by a matrix Θ, as we will consider a generalization of the function g discussed in Section  2.5, i.e. $g\left(\bar{v},\Theta \right)=\Theta h\left(\bar{v}\right)$, where each element i of vector $h\left(\bar{v}\right)$ depends only on $v_{i}$. This function corresponds to an assumption often made by models of feature extraction (Bell and Sejnowski, 1995, Olshausen and Field, 1995), that stimuli are formed by a linear combination of features.^5^ Moreover, such a function g can be easily computed as it is equal to an input to a layer of neurons from another layer with activity $h\left(\bar{v}\right)$ via connections with strength Θ.

We can state the negative free energy, analogously as for the simple model considered in Eq. (7) (TRY IT YOURSELF):

(37) $$F=\ln p\left(\bar{\phi }\right)+\ln p\left(\bar{u}|\bar{\phi }\right)=\frac{1}{2}\left(-\ln\left|\Sigma_{p}\right|-{\left(\bar{\phi }-{\bar{v}}_{p}\right)}^{T}\Sigma_{p}^{-1}\left(\bar{\phi }-{\bar{v}}_{p}\right)-\ln\left|\Sigma_{u}\right|-{\left(\bar{u}-g\left(\bar{\phi },\Theta \right)\right)}^{T}\Sigma_{u}^{-1}\left(\bar{u}-g\left(\bar{\phi },\Theta \right)\right)\right)+C\text{.}$$

Analogously as before, to find the vector of most likely values of features $\bar{\phi }$, we will calculate the gradient (vector of derivatives $\partial F/\partial \phi_{i}$) which we will denote by $\partial F/\partial \bar{\phi }$. We will use the elegant property that rules for computation of derivatives generalize to vectors and matrices. To get an intuition for these rules we recommend the following exercise that shows how the rule $\partial x^{2}/\partial x=2x$ generalizes to vectors.

Exercise 4 *Show that for any vector* $\bar{x}$*the gradient of function* $y={\bar{x}}^{T}\bar{x}$ *is equal to:* $\partial y/\partial \bar{x}=2\bar{x}$*.*

Using an analogous method as that in the solution to Exercise 4 (at the end of the paper) one can see that several other rules generalize as summarized in Table 1. These rules can be applied for symmetric matrices, but since Σ are covariance matrices, they are symmetric, so we can use the top two rules in Table 1 to compute the gradient of the negative free energy (TRY IT YOURSELF):

(38) $$\frac{\partial F}{\partial \bar{\phi }}=-\Sigma_{p}^{-1}\left(\bar{\phi }-{\bar{v}}_{p}\right)+\frac{{\partial g\left(\bar{\phi },\Theta \right)}^{T}}{\partial \bar{\phi }}\Sigma_{u}^{-1}\left(\bar{u}-g\left(\bar{\phi },\Theta \right)\right)\text{.}$$

In the above equation, terms appear which are generalizations of the prediction errors we defined for the simple models:

(39) $${\bar{\varepsilon }}_{p}=\Sigma_{p}^{-1}\left(\bar{\phi }-{\bar{v}}_{p}\right)$$

(40) $${\bar{\varepsilon }}_{u}=\Sigma_{u}^{-1}\left(\bar{u}-g\left(\bar{\phi },\Theta \right)\right)\text{.}$$

With the error terms defined, the equation describing the update of $\bar{\phi }$ becomes:

(41) $$\overset{˙}{\bar{\phi }}=-{\bar{\varepsilon }}_{p}+\frac{{\partial g\left(\bar{\phi },\Theta \right)}^{T}}{\partial \bar{\phi }}{\bar{\varepsilon }}_{u}\text{.}$$

The partial derivative term in the above equation is a matrix that contains in each entry with co-ordinates (i,j) the derivative of element i of vector $g\left(\bar{\phi },\Theta \right)$ over $\phi_{j}$. To see how the above equation simplifies for our choice of function g, it is helpful without loss of generality to consider a case of 2 features being estimated from 2 stimuli. Then:

(42) $$g\left(\bar{\phi },\Theta \right)=\Theta h\left(\bar{\phi }\right)=\left[\begin{array}{c} \theta_{1,1}h\left(\phi_{1}\right)+\theta_{1,2}h\left(\phi_{2}\right)\\ \theta_{2,1}h\left(\phi_{1}\right)+\theta_{2,2}h\left(\phi_{2}\right) \end{array}\right]\text{.}$$

Hence we can find the derivatives of elements of the above vector over the elements of vector $\bar{\phi }$:

(43) $$\frac{\partial g\left(\bar{\phi },\Theta \right)}{\partial \bar{\phi }}=\left[\begin{array}{cc} \theta_{1,1}h^{\prime }\left(\phi_{1}\right) & \theta_{1,2}h^{\prime }\left(\phi_{2}\right)\\ \theta_{2,1}h^{\prime }\left(\phi_{1}\right) & \theta_{2,2}h^{\prime }\left(\phi_{2}\right) \end{array}\right]\text{.}$$

Now we can see that Eq. (41) can be written as:

(44) $$\overset{˙}{\bar{\phi }}=-{\bar{\varepsilon }}_{p}+h^{\prime }\left(\bar{\phi }\right)\times \Theta^{T}{\bar{\varepsilon }}_{u}\text{.}$$

In the above equation × denotes element by element multiplication, so term $h^{\prime }\left(\bar{\phi }\right)\times \Theta^{T}{\bar{\varepsilon }}_{u}$ is a vector where its element i is equal to a product of $h^{\prime }\left(\phi_{i}\right)$ and element i of vector $\Theta^{T}{\bar{\varepsilon }}_{u}$. Analogously, as for the simple model, prediction errors could be computed by nodes with the following dynamics:

(45) $${\overset{˙}{\bar{\varepsilon }}}_{p}=\bar{\phi }-{\bar{v}}_{p}-\Sigma_{p}{\bar{\varepsilon }}_{p}$$

(46) $${\overset{˙}{\bar{\varepsilon }}}_{u}=\bar{u}-\Theta h\left(\bar{\phi }\right)-\Sigma_{u}{\bar{\varepsilon }}_{u}\text{.}$$

**Fig. 5:**
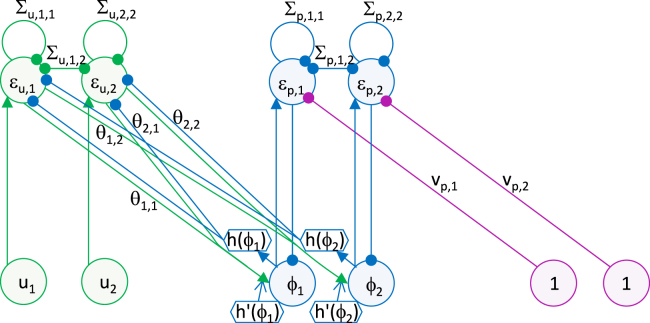
The architecture of the model inferring 2 features from 2 sensory stimuli. Notation as in Fig. 4(b). To help identify which connections are intrinsic and extrinsic to each level of hierarchy, the nodes and their projections in each level of hierarchy are shown in green, blue and purple respectively (in the online version). (For interpretation of the references to colour in this figure legend, the reader is referred to the web version of this article.)

It is easy to see that Eqs. (45)–(46) have fixed points at values given by Eqs. (39)–(40) by setting the left hand sides of Eqs. (45)–(46) to 0. The architecture of the network with the dynamics described by Eqs. (44), (45), (46) is shown in Fig. 5, and it is analogous to that in Fig. 4(b).

Analogously as for the simple model, one can also find the rules for updating parameters encoded in synaptic connections, which generalize the rules presented previously. In particular, using the top formula in Table 1 it is easy to see that:

(47) $$\frac{\partial F}{\partial {\bar{v}}_{p}}={\bar{\varepsilon }}_{p}\text{.}$$

Using the two bottom formulas in Table 1 one can find the rules for update of covariance matrices (TRY IT YOURSELF):

(48) $$\frac{\partial F}{\partial \Sigma_{p}}=\frac{1}{2}\left({\bar{\varepsilon }}_{p}{\bar{\varepsilon }}_{p}^{T}-\Sigma_{p}^{-1}\right)$$

(49) $$\frac{\partial F}{\partial \Sigma_{u}}=\frac{1}{2}\left({\bar{\varepsilon }}_{u}{\bar{\varepsilon }}_{u}^{T}-\Sigma_{u}^{-1}\right)\text{.}$$

The derivation of update of parameters Θ is a bit more tedious, but we show in Appendix B that:

(50) $$\frac{\partial F}{\partial \Theta }={\bar{\varepsilon }}_{u}h{\left(\bar{\phi }\right)}^{T}\text{.}$$

The above plasticity rules of Eqs. (47), (48), (49), (50) are Hebbian in the same sense they were for the simple model—for example Eq. (48) implies that $\Sigma_{p,i,j}$ should be updated proportionally to $\varepsilon_{p,i}\varepsilon_{p,j}$, i.e. to the product of activity of pre-synaptic and post-synaptic neurons. However, the rules of update of covariance matrices of Eqs. (48)–(49) contain matrix inverses $\Sigma^{-1}$. The value of each entry in matrix inverse depends on all matrix elements, so it is difficult how it can be “known” by a synapse that encodes just a single element. Nevertheless, we will show in Section  5 how the model can be extended to satisfy the constraint of local plasticity.

### 4.2 Introducing hierarchy

Sensory cortical areas are organized hierarchically, such that areas in lower levels of hierarchy (e.g. primary visual cortex) infer presence of simple features of stimuli (e.g. edges), on the basis of which the sensory areas in higher levels of hierarchy infer presence of more and more complex features. It is straightforward to generalize the model from 2 layers to multiple layers. In such generalized model the rules describing dynamics of neurons and plasticity of synapses remain exactly the same, and only notation has to be modified to describe presence of multiple layers of hierarchy.

We assume that the expected value of activity in one layer $v_{i}$ depends on the activity in the next layer $v_{i+1}$:

(51) $$E\left(\bar{u}\right)=g_{1}\left({\bar{v}}_{2},\Theta_{1}\right)$$

$$E\left({\bar{v}}_{2}\right)=g_{2}\left({\bar{v}}_{3},\Theta_{2}\right)$$

$$E\left({\bar{v}}_{3}\right)=\ldots \text{.}$$

To simplify the notation we could denote u by $v_{1}$, and then the likelihood of activity in layer i becomes:

(52) $$p\left({\bar{v}}_{i}|{\bar{v}}_{i+1}\right)=f\left({\bar{v}}_{i};g_{i}\left({\bar{v}}_{i+1},\Theta_{i}\right),\Sigma_{i}\right)\text{.}$$

**Fig. 6:**
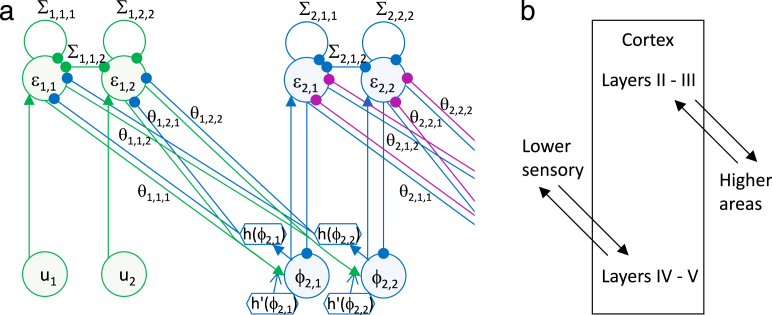
(a) The architecture of the model including multiple layers. For simplicity only the first two layers are shown. Notation as in Fig. 5. (b) Extrinsic connectivity of cortical layers.

In this model, $\Sigma_{i}$ parametrize the covariance between features in each level, and $\Theta_{i}$ parametrize how the mean value of features in one level depends on the next. Let us assume the same form of function g as before, i.e. $g_{i}\left({\bar{v}}_{i+1},\Theta_{i}\right)=\Theta_{i}h\left({\bar{v}}_{i+1}\right)$. By analogy to the model described in the previous subsection, one can see that inference of the features in all layers on the basis of sensory input can be achieved in the network shown in Fig. 6(a). In this network the dynamics of the nodes are described by:

(53) $$\overset{˙}{{\bar{\phi }}_{i}}=-{\bar{\varepsilon }}_{i}+h^{\prime }\left({\bar{\phi }}_{i}\right).*{\Theta_{i-1}}^{T}{\bar{\varepsilon }}_{i-1}$$

(54) $${\overset{˙}{\bar{\varepsilon }}}_{i}={\bar{\phi }}_{i}-\Theta_{i}h\left({\bar{\phi }}_{i+1}\right)-\Sigma_{i}{\bar{\varepsilon }}_{i}\text{.}$$

Furthermore, by analogy to the previous section, the rules for modifying synaptic connections in the model become:

(55) $$\frac{\partial F}{\partial \Sigma_{i}}=\frac{1}{2}\left({\bar{\varepsilon }}_{i}{\bar{\varepsilon }}_{i}^{T}-\Sigma_{i}^{-1}\right)$$

(56) $$\frac{\partial F}{\partial \Theta_{i}}={\bar{\varepsilon }}_{i}h{\left({\bar{\phi }}_{i+1}\right)}^{T}\text{.}$$

The hierarchical structure of the model in Fig. 6(a) parallels the hierarchical structure of the cortex. Furthermore, it is worth noting that different layers within the cortex communicate with higher and lower sensory areas (as illustrated schematically in Fig. 6(b)), which parallel the fact that different nodes in the model communicate with other levels of hierarchy (Fig. 6(a)).

## 5 Local plasticity

The plasticity rules for synapses encoding matrix Σ (describing the variance and co-variance of features or sensory inputs) introduced in the previous section (Eqs. (48), (49), (55)) include terms equal to the matrix inverse $\Sigma^{-1}$. Computing each element of the inverse $\Sigma^{-1}$ requires not only the knowledge of the corresponding element of Σ, but also of other elements. For example, in a case of 2-dimensional vector $\bar{u}$, the update rule for the synaptic connection encoding $\Sigma_{u,1,1}$ (Eq. (49)) requires the computation of $\Sigma_{u,1,1}^{-1}=\Sigma_{u,2,2}/\left|\Sigma_{u}\right|$. Hence the change of synaptic weight $\Sigma_{u,1,1}$ depends on the value of the weight $\Sigma_{u,2,2}$, but these are the weights of connections between different neurons (see Fig. 5), thus the update rule violates the principle of the local plasticity stated in the Introduction. Nevertheless, in this section we show that by slightly modifying the architecture of the network computing prediction errors, the need for computing matrix inverses in the plasticity rules disappears. In other words, we present an extension of the model from the previous section in which learning the values of parameters Σ satisfies the constraint of local plasticity. To make the description as easy to follow as possible, we start with considering the case of single sensory input and single feature on each level, and then generalize it to increased dimension of inputs and features.

### 5.1 Learning variance of a single prediction error node

Instead of considering the whole model we now focus on computations in a single node computing prediction error. In the model we wish the prediction error on each level to converge to:

(57) $$\varepsilon_{i}=\frac{\phi_{i}-g_{i}\left(\phi_{i+1}\right)}{\Sigma_{i}}\text{.}$$

In the above equation $\Sigma_{i}$ is the variance of feature $\phi_{i}$ (around the mean predicted by the level above):

(58) $$\Sigma_{i}=\langle {\left(\phi_{i}-g_{i}\left(\phi_{i+1}\right)\right)}^{2}\rangle \text{.}$$

**Fig. 7:**
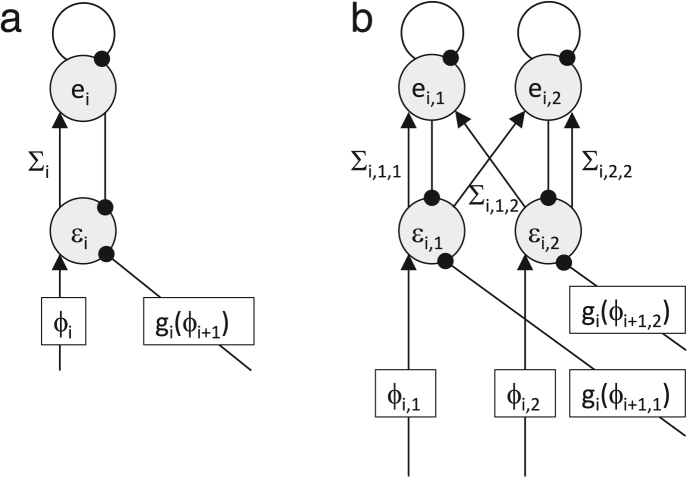
Prediction error networks that can learn the uncertainty parameter with local plasticity. Notation as in Fig. 4(b). (a) Single node. (b) Multiple nodes for multidimensional features.

A sample architecture of the model that can achieve this computation with local plasticity is shown in Fig. 7(a). It includes an additional inhibitory inter-neuron $e_{i}$ which is connected to the prediction error node, and receives input from it via the connection with weight encoding $\Sigma_{i}$. The dynamics of this model is described by the following set of equations:

(59) $$\overset{˙}{\varepsilon_{i}}=\phi_{i}-g_{i}\left(\phi_{i+1}\right)-e_{i}$$

(60) $$\overset{˙}{e_{i}}=\Sigma_{i}\varepsilon_{i}-e_{i}\text{.}$$

The levels of activity at the fixed point can be found by setting the left hand sides of Eqs. (59)–(60) to 0 and solving the resulting set of simultaneous equations (TRY IT YOURSELF):

(61) $$\varepsilon_{i}=\frac{\phi_{i}-g_{i}\left(\phi_{i+1}\right)}{\Sigma_{i}}$$

(62) $$e_{i}=\phi_{i}-g_{i}\left(\phi_{i+1}\right)\text{.}$$

Thus we see that the prediction error node has a fixed point at the desired value (cf. Eq. (57)). Let us now consider the following rule for plasticity of the connection encoding $\Sigma_{i}$:

(63) $$\Delta \Sigma_{i}=\alpha \left(\varepsilon_{i}e_{i}-1\right)\text{.}$$

According to this rule the weight is modified proportionally to the product of activities of pre-synaptic and post-synaptic neurons decreased by a constant, with a learning rate α. To analyse to what values this rule converges, we note that the expected change is equal to 0 when:

(64) $$\langle \varepsilon_{i}e_{i}-1\rangle =0\text{.}$$

Substituting Eqs. (61)–(62) into the above equation and rearranging terms we obtain:

(65) $$\frac{\langle {\left(\phi_{i}-g_{i}\left(\phi_{i+1}\right)\right)}^{2}\rangle }{\Sigma_{i}}=1\text{.}$$

Solving the above equation for $\Sigma_{i}$ we obtain Eq. (58). Thus in summary the network in Fig. 7(a) computes the prediction error and learns the variance of the corresponding feature with a local Hebbian plasticity rule. To gain more intuition for how this model works we suggest the following exercise.

Exercise 5 *Simulate learning of variance* $\Sigma_{i}$ *over trials. For simplicity, only simulate the network described by Eqs.*   (59)*–*(60)*, and assume that variables* ϕ *are constant. On each trial generate input* $\phi_{i}$ *from a normal distribution with mean*  5  *and variance*  2*, while set* $g_{i}\left(\phi_{i+1}\right)=5$ *(so that the upper level correctly predicts the mean of* $\phi_{i}$ *). Simulate the network for*  20  *time units, and then update weight* $\Sigma_{i}$ *with learning rate* α=0.01*. Simulate*  1000  *trials and plot how* $\Sigma_{i}$ *changes across trials.*

**Fig. 8:**
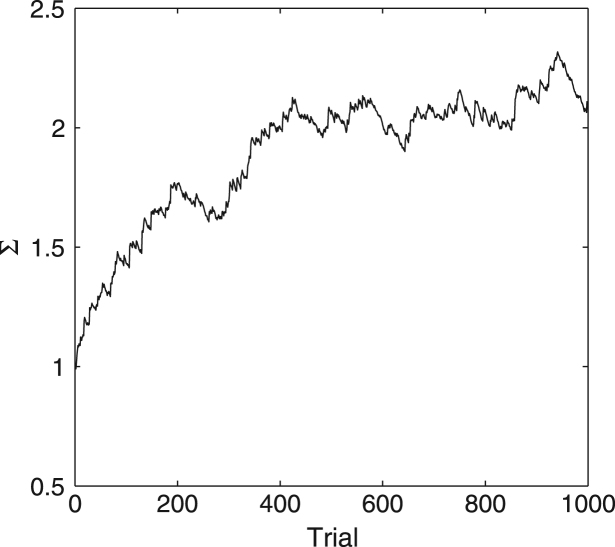
Changes in estimated variance during learning in Exercise 5.

The results of simulations are shown in Fig. 8, and they illustrate that the synaptic weight $\Sigma_{i}$ approaches the vicinity of the variance of $\phi_{i}$.

It is also worth adding that $\varepsilon_{i}$ in the model described by Eqs. (59)–(60) converges to the prediction error (Eq. (61)), when one assumes that ϕ are constant or change on much slower time-scale than $\varepsilon_{i}$ and $e_{i}$. This convergence takes place because the fixed point of the model is stable, which can be shown using the standard dynamical systems theory (Strogatz, 1994). In particular, since Eqs. (59)–(60) only contain linear functions of variables $\varepsilon_{i}$ and $e_{i}$, their solution has a form of exponential functions of time t, e.g. $\varepsilon_{i}\left(t\right)=c\exp\left(\lambda t\right)+\varepsilon_{i}^{*}$, where c and λ are constants, and $\varepsilon_{i}^{*}$ is the value at the fixed point. The sign of λ determines the stability of the fixed point: when λ<0, the exponential term decreases with time, and $\varepsilon_{i}$ converges to the fixed point, while if λ>0, the fixed point is unstable. The values of λ are equal to the eigenvalues of the matrix in the equation below (Strogatz, 1994), which rewrites Eqs. (59)–(60) in a vector form:

(66) $$\left[\begin{array}{c} {\overset{˙}{\varepsilon }}_{i}\\ {\overset{˙}{e}}_{i} \end{array}\right]=\left[\begin{array}{cc} 0 & -1\\ \Sigma_{i} & -1 \end{array}\right]\left[\begin{array}{c} \varepsilon_{i}\\ e_{i} \end{array}\right]+\left[\begin{array}{c} \phi_{i}-g_{i}\left(\phi_{i+1}\right)\\ 0 \end{array}\right]\text{.}$$

To show that the eigenvalues of the matrix in the above equation are negative we use the property that sum of eigenvalues is equal to the trace and the product to the determinant. The trace and determinant of this matrix are −1 and $\Sigma_{i}$, respectively. Since the sum of eigenvalues is negative and their product positive, both eigenvalues are negative, so the system is stable.

### 5.2 Learning the covariance matrix

The model described in the previous subsection scales up to larger dimension of features and sensory inputs. The architecture of the scaled up network is shown in Fig. 7(b), and its dynamics is described by the following equations:

(67) $$\overset{˙}{{\bar{\varepsilon }}_{i}}={\bar{\phi }}_{i}-g_{i}\left({\bar{\phi }}_{i+1}\right)-{\bar{e}}_{i}$$

(68) $$\overset{˙}{{\bar{e}}_{i}}=\Sigma_{i}{\bar{\varepsilon }}_{i}-{\bar{e}}_{i}\text{.}$$

Analogously as before, we can find the fixed point by setting the left hand side of the equation to 0:

(69) $${\bar{\varepsilon }}_{i}=\Sigma_{i}^{-1}\left({\bar{\phi }}_{i}-g_{i}\left({\bar{\phi }}_{i+1}\right)\right)$$

(70) $${\bar{e}}_{i}={\bar{\phi }}_{i}-g_{i}\left({\bar{\phi }}_{i+1}\right)\text{.}$$

Thus we can see that nodes ε have fixed points at the values equal to the prediction errors. We can now consider a learning rule analogous to that in the previous subsection:

(71) $$\Delta \Sigma_{i}=\alpha \left({\bar{\varepsilon }}_{i}{\bar{e}}_{i}^{T}-1\right)\text{.}$$

To find the values to vicinity of which the above rule may converge, we can find the value of $\Sigma_{i}$ for which the expected value of the right hand side of the above equation is equal to 0:

(72) $$\langle {\bar{\varepsilon }}_{i}{\bar{e}}_{i}^{T}-1\rangle =0\text{.}$$

Substituting Eqs. (69)–(70) into the above equation, and solving for $\Sigma_{i}$ we obtain (TRY IT YOURSELF):

(73) $$\Sigma_{i}=\langle \left({\bar{\phi }}_{i}-g_{i}\left({\bar{\phi }}_{i+1}\right)\right){\left({\bar{\phi }}_{i}-g_{i}\left({\bar{\phi }}_{i+1}\right)\right)}^{T}\rangle \text{.}$$

We can see that the learning rule has a stochastic fixed point at the values corresponding to the covariance matrix. In summary, the nodes in network described in this section have fixed points at prediction errors and can learn the covariance of the corresponding features, thus the proposed network may substitute the prediction error nodes in the model shown in Fig. 6, and the computation will remain the same. But importantly in the proposed network the covariance is learnt with local plasticity involving simple Hebbian learning.

## 6 Discussion

In this paper we presented the model of perception and learning in neural circuits based on the free-energy framework. This model extends the predictive coding model (Rao & Ballard, 1999) in that it represents and learns not only mean values of stimuli or features, but also their variances, which gives the model several new computational capabilities, as we now discuss.

First, the model can weight incoming sensory information by their reliability. This property arises in the model, because the prediction errors are normalized by dividing them by the variance of noise. Thus the more noisy is a particular dimension of the stimulus, the smaller the corresponding prediction error, and thus lower its influence on activity on other neurons in the network.

**Fig. 9:**
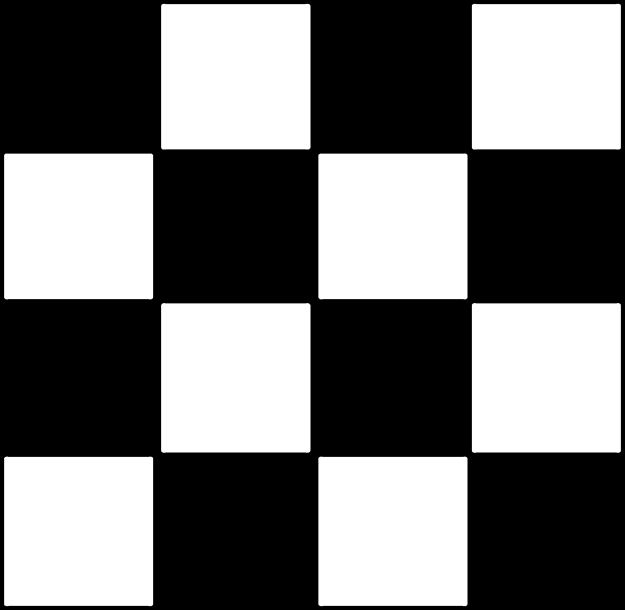
An example of a texture.

Second, the model can learn properties of features encoded in covariance of sensory input. An example of such feature is texture, which can be efficiently recognized on the basis of covariance, irrespectively of translation (Harwood, Ojala, Pietikäinen, Kelman, & Davis, 1995). To get an intuition for this property, let us consider an example of checker-board texture (Fig. 9). Please note that adjacent nodes have always opposite colour–corresponding to negative covariance, while the diagonal nodes have the same colour–corresponding to positive covariance.

Third, the attentional modulation can be easily implemented in the model by changing the variance associated with the attended features (Feldman & Friston, 2010). Thus for example, attending to feature i at level j of the hierarchy can be implemented by decreasing synaptic weight $\Sigma_{j,i,i}$, or inhibiting node $e_{j,i}$ in case of the model described in Section  5, which will result in a larger effect of the node encoding this feature on the activity in the rest of the network.

In this paper we included description of the modified or extended version of the model with local computation and plasticity to better illustrate how computation proposed by the free-energy framework can be implemented in neural circuits. However, it will be necessary in the future to numerically evaluate the efficiency of learning in the proposed model and the free-energy framework in general. Existing models of feature extraction (Bell and Sejnowski, 1997, Bogacz et al., 2001, Olshausen and Field, 1995) and predictive coding (Rao & Ballard, 1999) have been shown to be able to find features efficiently and reproduce the receptive fields of neurons in the primary visual cortex when trained with natural images. It would be interesting to explicitly test in simulations if the model based on the free-energy framework can equally efficiently extract features from natural stimuli and additionally learn the variance and covariance of features.

We have also demonstrated that if the dynamics within the nodes computing prediction errors takes place on a time-scale much faster than in the whole network, these nodes converge to stable fixed points. It is also worth noting that under the assumption of separation of time scales, the nodes computing ϕ also converge to a stable fixed point, because variables ϕ converge to the values that maximize function F. It would be interesting to investigate how to ensure that the model converges to desired values (rather than engaging into oscillatory behaviour) also when one considers a more realistic case of time-scales not being fully separated.

In summary, in this paper we presented the free-energy theory, which offers a powerful framework for describing computations performed by the brain during perception and learning. The appeal of the similarity in the organization of networks suggested by this theory and observed in the brain invites attempts to map the currently relatively abstract models on details of cortical micro-circuitry, i.e. to map different elements of the model on different neural populations within the cortex. For example, Bastos et al. (2012) compared a more recent version of the model (Friston, 2008) with the details of the cortical organization. Such comparisons of the models with biological circuits are likely to lead to iterative refinement of the models.

Even if the free-energy framework does describe cortical computation, the mapping between the variables in the model and the elements of neural circuit may not be “clean” but rather “messy” i.e. each model variable or parameter may be represented by multiple neurons or synapses. The particular implementation of the framework in the cortical circuit may be influenced by other constraints the evolutionary pressure optimizes such as robustness to damage, energy efficiency, speed of processing, etc. In any case, the comparison of predictions of theoretical framework like the free-energy with experimental data offers hope for understanding the cortical micro-circuits.

## Figures and Tables

**Fig. 10:**
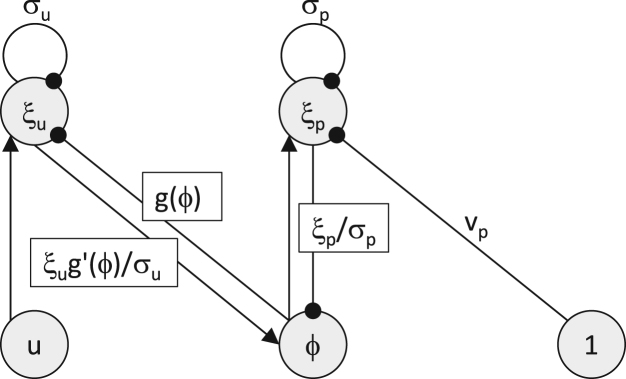
The architectures of the original model performing simple perceptual inference. Notation as in Fig. 3.

**Figure:**
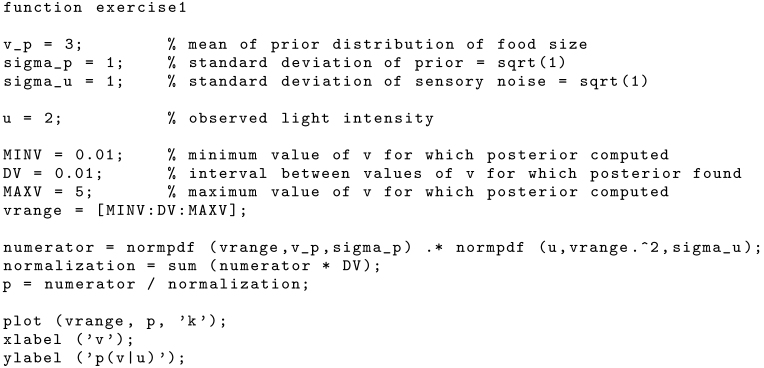


**Figure:**
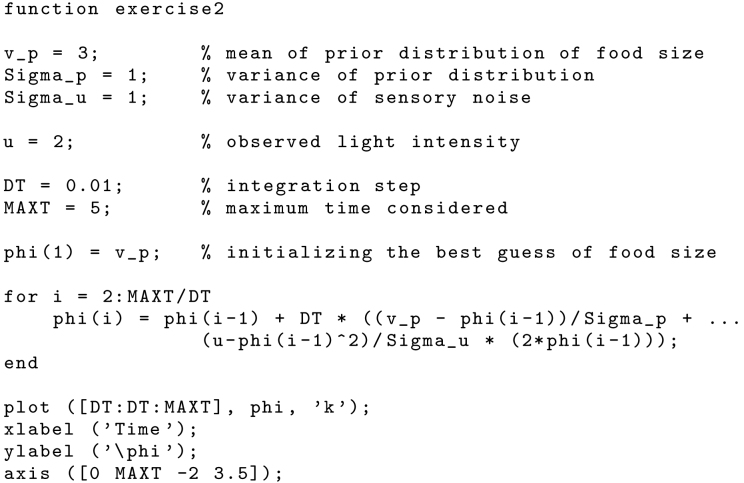


**Figure:**
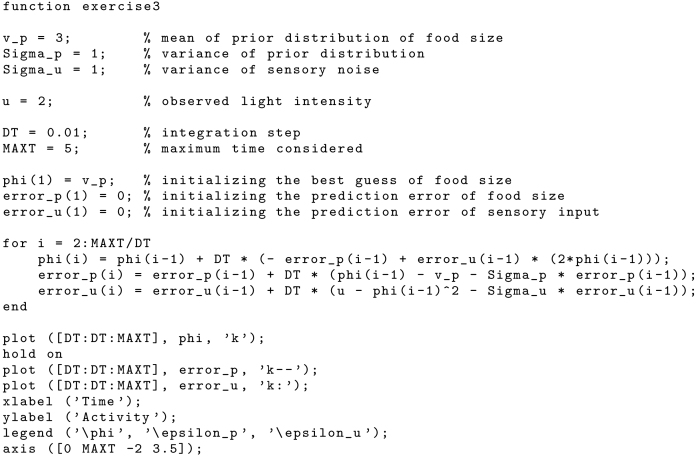


**Figure:**
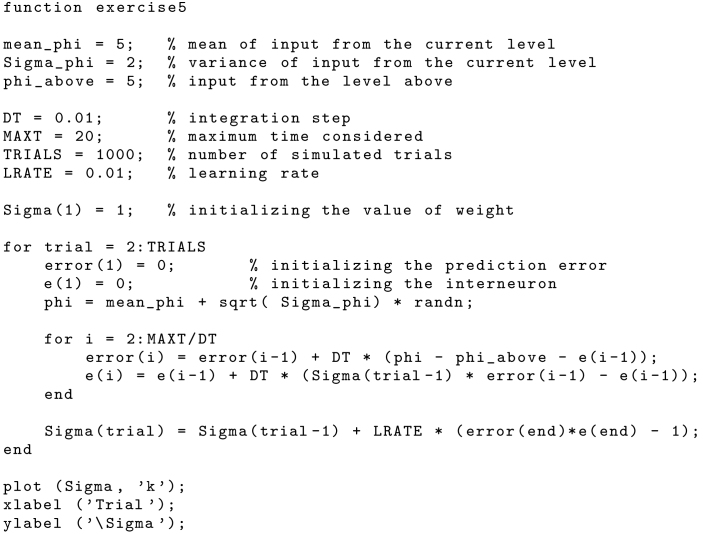


**Table 1:** Rules for computation of derivatives. A denotes a symmetric matrix.

Original rule	Generalization to matrices
$\frac{\partial ax^{2}}{\partial x}=2ax$	$\frac{\partial {\bar{x}}^{T}A\bar{x}}{\partial \bar{x}}=2A\bar{x}$
if z=f(y), y=g(x), then $\frac{\partial z}{\partial x}=\frac{\partial y}{\partial x}\frac{\partial z}{\partial y}$	if $z=f\left(\bar{y}\right)$, $\bar{y}=g\left(\bar{x}\right)$, then $\frac{\partial z}{\partial \bar{x}}={\left(\frac{\partial \bar{y}}{\partial \bar{x}}\right)}^{T}\frac{\partial z}{\partial \bar{y}}$
$\frac{\partial \ln a}{\partial a}=\frac{1}{a}$	$\frac{\partial \ln\left|A\right|}{\partial A}=A^{-1}$
$\frac{\partial \frac{x^{2}}{a}}{\partial a}=-\frac{x^{2}}{a^{2}}$	$\frac{\partial {\bar{x}}^{T}A^{-1}\bar{x}}{\partial A}=-\left(A^{-1}\bar{x}\right){\left(A^{-1}\bar{x}\right)}^{T}$

## Appendix A The original neural implementation

In the original model (Friston, 2005), the prediction errors were defined in a slightly different way:

(74) $$\xi_{p}=\frac{\phi -v_{p}}{\sigma_{p}}$$

(75) $$\xi_{u}=\frac{u-g\left(\phi \right)}{\sigma_{u}}\text{.}$$

In the above equations, $\sigma_{p}=\sqrt{\Sigma_{p}}$, and $\sigma_{u}=\sqrt{\Sigma_{u}}$, i.e. $\sigma_{p}$ and $\sigma_{u}$ denote the standard deviations of distributions p(v) and p(u|v) respectively. With the prediction error terms defined in this way, the negative free energy computed in Eq. (7) can be written as:

(76) $$F=-\ln\sigma_{p}-\frac{1}{2}\xi_{p}^{2}-\ln\sigma_{u}-\frac{1}{2}\xi_{u}^{2}+C_{2}\text{.}$$

The dynamics of variable ϕ is proportional to the derivative of the above equation over ϕ:

(77) $$\overset{˙}{\phi }=\frac{\xi_{u}g^{\prime }\left(\phi \right)}{\sigma_{u}}-\frac{\varepsilon_{p}}{\sigma_{p}}\text{.}$$

Analogously as in Section  2.3, the prediction errors defined in Eqs. (74)–(75) could be computed in the nodes with the following dynamics:

(78) $$\overset{˙}{\xi_{p}}=\phi -v_{p}-\sigma_{p}\xi_{p}$$

(79) $$\overset{˙}{\xi_{u}}=u-g\left(\phi \right)-\sigma_{u}\xi_{u}\text{.}$$

The architecture of the model described by Eqs. (77), (78), (79) is shown in Fig. 10. It is similar to that in Fig. 3, but differs in the information received by node ϕ (we will discuss this difference in more detail at the end of this Appendix).

Analogously as before, we can find the rules describing synaptic plasticity in the model, by calculating the derivatives of F (Eq. (76)) over $v_{p}$, $\sigma_{p}$ and $\sigma_{u}$ (TRY IT YOURSELF):

(80) $$\frac{\partial F}{\partial v_{p}}=\xi_{p}\sigma_{p}^{-1}$$

(81) $$\frac{\partial F}{\partial \sigma_{p}}=\left(\xi_{p}^{2}-1\right)\sigma_{p}^{-1}$$

(82) $$\frac{\partial F}{\partial \sigma_{u}}=\left(\xi_{u}^{2}-1\right)\sigma_{u}^{-1}\text{.}$$

The original model does not satisfy the constraint of local computation stated in the Introduction, because the node computing ϕ receives the input from prediction error nodes scaled by parameters σ (see Fig. 10), but the parameters σ are not encoded in the connections between node ϕ and the prediction error nodes, but instead in the connections among the prediction error neurons. Nevertheless, we have shown in Section  2.3 that by just changing the way in which prediction errors are normalized the computation in the model becomes local.

## Appendix B Derivation of plasticity rule for connections between layers

This Appendix derives the rule for update of Θ given in Eq. (50). In order to use the two top formulas in Table 1 we have to reshape the matrix Θ into a vector. To avoid death by notation, without loss of generality, let us consider the case of 2 dimensional stimuli and features. So let us define the vector of parameters: $\bar{\theta }=\left[\theta_{1,1},\theta_{1,2},\theta_{2,1},\theta_{2,2}\right]$. Now, using two top formulas in Table 1 one can find that:

(83) $$\frac{\partial F}{\partial \bar{\theta }}=\frac{{\partial g\left(\bar{\phi },\Theta \right)}^{T}}{\partial \bar{\theta }}{\bar{\varepsilon }}_{u}\text{.}$$

From Eq. (42) we find:

(84) $$\frac{\partial g{\left(\bar{\phi },\Theta \right)}^{T}}{\partial \bar{\theta }}=\left[\begin{array}{cccc} h\left(\phi_{1}\right) & h\left(\phi_{2}\right) & 0 & 0\\ 0 & 0 & h\left(\phi_{1}\right) & h\left(\phi_{2}\right) \end{array}\right]\text{.}$$

We can now evaluate the right hand side of Eq. (83):

(85) $$\frac{{\partial g\left(\bar{\phi },\Theta \right)}^{T}}{\partial \bar{\theta }}{\bar{\varepsilon }}_{u}=\left[\begin{array}{cc} h\left(\phi_{1}\right) & 0\\ h\left(\phi_{2}\right) & 0\\ 0 & h\left(\phi_{1}\right)\\ 0 & h\left(\phi_{2}\right) \end{array}\right]\left[\begin{array}{c} \varepsilon_{u,1}\\ \varepsilon_{u,2} \end{array}\right]=\left[\begin{array}{c} h\left(\phi_{1}\right)\varepsilon_{u,1}\\ h\left(\phi_{2}\right)\varepsilon_{u,1}\\ h\left(\phi_{1}\right)\varepsilon_{u,2}\\ h\left(\phi_{2}\right)\varepsilon_{u,2} \end{array}\right]\text{.}$$

Reshaping the right hand side of the above equation into a matrix, we can see how it can be decomposed into the product of vectors in Eq. (50):

(86) $$\left[\begin{array}{cc} h\left(\phi_{1}\right)\varepsilon_{u,1} & h\left(\phi_{2}\right)\varepsilon_{u,1}\\ h\left(\phi_{1}\right)\varepsilon_{u,2} & h\left(\phi_{2}\right)\varepsilon_{u,2} \end{array}\right]=\left[\begin{array}{c} \varepsilon_{u,1}\\ \varepsilon_{u,2} \end{array}\right]\left[\begin{array}{cc} h\left(\phi_{1}\right) & h\left(\phi_{2}\right) \end{array}\right]\text{.}$$

## Solutions to exercises

### Exercise 4

It is easiest to consider a vector of two numbers (analogous can be shown for longer vectors):

(87) $$\bar{x}=\left[\begin{array}{c} x_{1}\\ x_{2} \end{array}\right]\text{.}$$

Then $y={\bar{x}}^{T}\bar{x}=x_{1}^{2}+x_{2}^{2}$, so the gradient is equal to:

(88) $$\frac{\partial y}{\partial \bar{x}}=\left[\begin{array}{c} \frac{\partial y}{\partial x_{1}}\\ \frac{\partial y}{\partial x_{2}} \end{array}\right]=\left[\begin{array}{c} 2x_{1}\\ 2x_{2} \end{array}\right]=2\bar{x}\text{.}$$

## Footnotes

1 In the original model (Friston, 2005) the prediction errors were normalized slightly differently as explained in Appendix A.

2 The original model does not provide details on the dynamics of the nodes computing prediction error, but we consider sample description of their dynamics to illustrate how these nodes can perform their computation.

3 In the original model, variables $\lambda =\sqrt{\Sigma }-1$ were defined, and these variables were encoded in the synaptic connections. Formally, this constraint is known as a hyperprior. This is because the variance or precision parameters are often referred to mathematically as hyperparameters. Whenever we place constraints on hyperparameters we necessarily invoke hyperpriors.

4 Although this case has not been discussed by Friston (2005), it was discussed by Rao and Ballard (1999).

5 In the model of Rao and Ballard (1999) the sparse coding was achieved through introduction of additional prior expectation that most $\phi_{i}$ are close to 0, but the sparse coding can also be achieved by choosing a shape of function h such that $h\left(v_{i}\right)$ are mostly close to 0, but only occasionally significantly different from zero (Friston, 2008).
